# Outcomes of intraocular lens exchange with combined pars plana vitrectomy and retropupillary iris-claw lens fixation

**DOI:** 10.1186/s40942-025-00791-1

**Published:** 2026-01-12

**Authors:** Denise Pardini, Carolina Hilgert, Olívia Pereira Kiappe, Talita Virgínia Fernandes de Oliveira, Jorge Henrique Cavalcante Tavares, Marina Roizenblatt, Michel Eid Farah, Maurício Maia, André Maia

**Affiliations:** 1 Department of Ophthalmology, Federal University of São Paulo, 822, Botucatu St. Vila Clementino, São Paulo, SP Brazil; 2 Vision Institute, Instituto Paulista de Estudos e Pesquisas em Oftalmologia (IPEPO), 1083, Borges Lagoa St. Vila Clementino, São Paulo, Brazil; 3 Retina Clinic, 1881, Estados Unidos St. Jardim América, São Paulo, Brazil; 4 75, Gonçalves Dias St. Funcionários., Belo Horizonte, MG Brazil

**Keywords:** Aphakia, Intraocular lens exchange, Retropupillary iris‑claw intraocular lens, Pars plana vitrectomy, Secondary implants

## Abstract

**Purpose:**

To evaluate the surgical outcomes of intraocular lens (IOL) exchange combining pars plana vitrectomy (PPV) with retropupillary iris‑claw IOL (ICIOL) fixation.

**Methods:**

This was a retrospective series of 33 eyes of 32 patients undergoing PPV and secondary retropupillary ICIOL implantation. The primary outcomes included the best‑corrected visual acuity (BCVA), refraction (spherical equivalent [SE], refractive astigmatism), surgically induced astigmatism (SIA) and prediction error. Secondary outcomes included pachymetry, intraocular pressure, surgical time, and complications.

**Results:**

The mean BCVA improved from the baseline logarithm of the minimum angle of resolution (logMAR) visual acuity of 0.73 ± 0.55 to 0.18 ± 0.24 at 6 months (*p* < 0.001). The mean final SE was − 0.40 ± 0.97 diopters (D) (*p* = 0.001), mean refractive astigmatism 1.36 ± 1.30 D (*p* = 0.955), mean SIA 1.39 ± 0.93 D, and mean prediction error − 0.28 ± 0.99 D. The mean pachymetry was stable (preoperative 562 ± 35.09 μm; postoperative 558 ± 42.15 μm; *p* = 0.122). The mean IOP was 15.85 ± 4.89 mmHg preoperatively and 14.72 ± 4.10 mmHg postoperatively (*p* = 0.563). The mean surgical time was 25.3 ± 4.5 min. Postoperative events included hyphema in 9.1%, cystoid macular edema in 6%, ocular hypertension in 6%, and IOL dislocation in 6%.

**Conclusions:**

IOL exchange with combined PPV and retropupillary ICIOL fixation achieved favorable visual and refractive outcomes with a short operating time and low complication rates, supporting this approach as an efficient option for eyes without capsular support.

**Supplementary Information:**

The online version contains supplementary material available at 10.1186/s40942-025-00791-1.

## Background

Intraocular lens (IOL) exchange remains challenging, particularly in the absence of capsular support. Common indications include dislocation, refractive error, IOL opacification, and ocular inflammation [1, 2]. The first step is explantation, either cutting and removing the IOL through a small corneal incision or extracting it via a larger corneal or scleral incision [3]. The second, and often more critical step, is selecting the method for secondary fixation or, alternatively, leaving the eye aphakic and correcting the refraction with a contact lens.

Available options for secondary IOL placement include: an anterior-chamber IOL (ACIOL) supported at the angle or fixated to the anterior iris surface and a posterior-chamber IOL (PCIOL) fixated behind the iris either to the posterior iris surface or the sclera. There is no consensus on the superiority of either method [2].

While ACIOL implantation can be faster and technically straightforward compared with iris or scleral fixation, it carries risks such as endothelial cell loss, cystoid macular edema (CME), glaucoma, and pupil distortion [4]. Posterior-chamber techniques using iris or scleral fixation achieve good visual outcomes, but complications may include suture exposure or breakage, vitreous or choroidal hemorrhage, intraocular pressure (IOP) fluctuations, retinal detachment, and chronic macular edema, the last two often related to vitreous traction [5–8].

Combining pars plana vitrectomy (PPV) with retropupillary iris‑claw IOL (ICIOL) implantation may mitigate vitreous‑related complications and reduce endothelial cell loss while providing stable support [9]. The Artisan Aphakia IOL (model 205, Ophtec, Boca Raton, FL, USA), a rigid polymethyl methacrylate lens designed for anterior or posterior iris fixation, has a 5.4‑mm optic and 8.5‑mm haptics with a broad dioptric range (+ 2.0 to + 30.0 diopters [D] in 1.0‑D steps and + 14.5 to + 24.5 D in 0.5‑D steps) [10].

Although several published studies have reported favorable outcomes of secondary ICIOL implantation, few have provided a comprehensive refractive analysis, including assessment of surgically induced astigmatism (SIA), prediction error relative to biometric calculation, and pre- and postoperative corneal topography. In this study, we evaluated the refractive and anatomic outcomes of IOL exchange using combined PPV and retropupillary Artisan IOL fixation.

## Methods

This retrospective case series included patients who underwent IOL exchange with combined PPV and retropupillary iris-claw IOL fixation between June 2017 and November 2020. The Ethics Committee of the Federal University of São Paulo approved the study, which was conducted according to the Declaration of Helsinki.

The inclusion criteria were patients with dislocated or opacified IOLs undergoing an exchange with retropupillary ICIOL fixation combined with PPV. In cases of IOL opacification without frank dislocation, capsular and zonular support was considered inadequate or unstable after removal of the original IOL, precluding safe in-the-bag or sulcus IOL implantation. Therefore, a retropupillary iris-claw IOL was selected as a stable secondary fixation option independent of capsular support. The exclusion criteria included a follow-up shorter than 6 months, incomplete refractive data, or pre-fixation macular or corneal disease.

The collected data included demographics, ocular history, previous surgeries, follow-up time, surgical indications, intraoperative complications, surgical time, pre- and post-fixation best-corrected visual acuity (BCVA) (Snellen converted to the logarithm of the minimum angle of resolution [logMAR]), refraction (spherical equivalent [SE], refractive astigmatism), corneal topography and tomography, biometry, pachymetry, IOP, and postoperative complications.

Biometric IOL power calculations were performed using optical biometry (Pentacam, Wetzlar, Germany) or immersion ultrasound (Aviso, Quantel Medical, Cournon d’Auvergne, France) with the SRK/T formula. The A-constant used was 116.9 for retropupillary implantation [9, 11]. Patients were examined on day 1, week 1, and months 1, 3, and 6 postoperatively, with additional visits as needed. Hypotony was defined as IOP ≤ 6 mmHg, ocular hypertension as IOP ≥ 25 mmHg on at least one postoperative visit requiring initiation or escalation of topical hypotensive therapy, corneal edema as a 5% or greater increase in central corneal thickness relative to baseline persisting 15 days or more and/or corneal haze lasting 15 days or more, and CME as new postoperative edema confirmed by optical coherence tomography (Zeiss, Cirrus 6000).

The prediction error was defined as the difference between the target refraction and the final SE. SIA was calculated using the Hill online calculator [12]. Statistical analyses used mixed linear models to compare outcomes across time points, Kolmogorov–Smirnov testing for normality, and McNemar testing for categorical variables. Significance was set at *p* < 0.05. Analyses were performed using SPSS 20.0 (SPSS Inc., Chicago, IL, USA) and STATA 18 (Stata Corp., College Station, TX, USA).

## Surgical technique

The same experienced surgeon (AM) performed all surgeries. A 5.5-mm scleral tunnel was created 2 mm from the limbus after a superior peritomy, without initially entering the anterior chamber. A standard 23-gauge three-port PPV (Constellation System, Alcon Laboratories, Ft. Worth, TX, USA) was performed, including core vitrectomy and induction of posterior vitreous detachment.

IOL explantation was performed in all 33 eyes included in the study. The surgical technique varied according to the IOL position, i.e., either displaced into the posterior vitreous cavity or subluxated into the anterior vitreous or posterior chamber. In the first case, the lens was grasped with end-gripping forceps, brought into the anterior chamber, and removed through the scleral tunnel. In the second case, a spatula was placed behind the IOL to support it while it was mobilized into the anterior chamber using an IOL chopper and subsequently extracted through the scleral tunnel. Any residual vitreous, capsular remnants, or fibrous tissue interfering with IOL positioning was excised.

The retropupillary iris-claw IOL (Artisan Aphakia model 205) was introduced into the anterior chamber and gently positioned behind the iris plane. The IOL was enclavated between the mid-peripheral iris and pupillary edge to avoid pupil ovalization. Carbachol was instilled to achieve miosis, and stability was verified by central tapping. The scleral tunnel was sutured with 10 − 0 nylon if necessary, and the conjunctiva was closed with 8 − 0 Vicryl or tissue glue (supplementary video 1). A standard postoperative regimen of topical antibiotics and corticosteroids was prescribed.

## Results

**Fig. 1:**
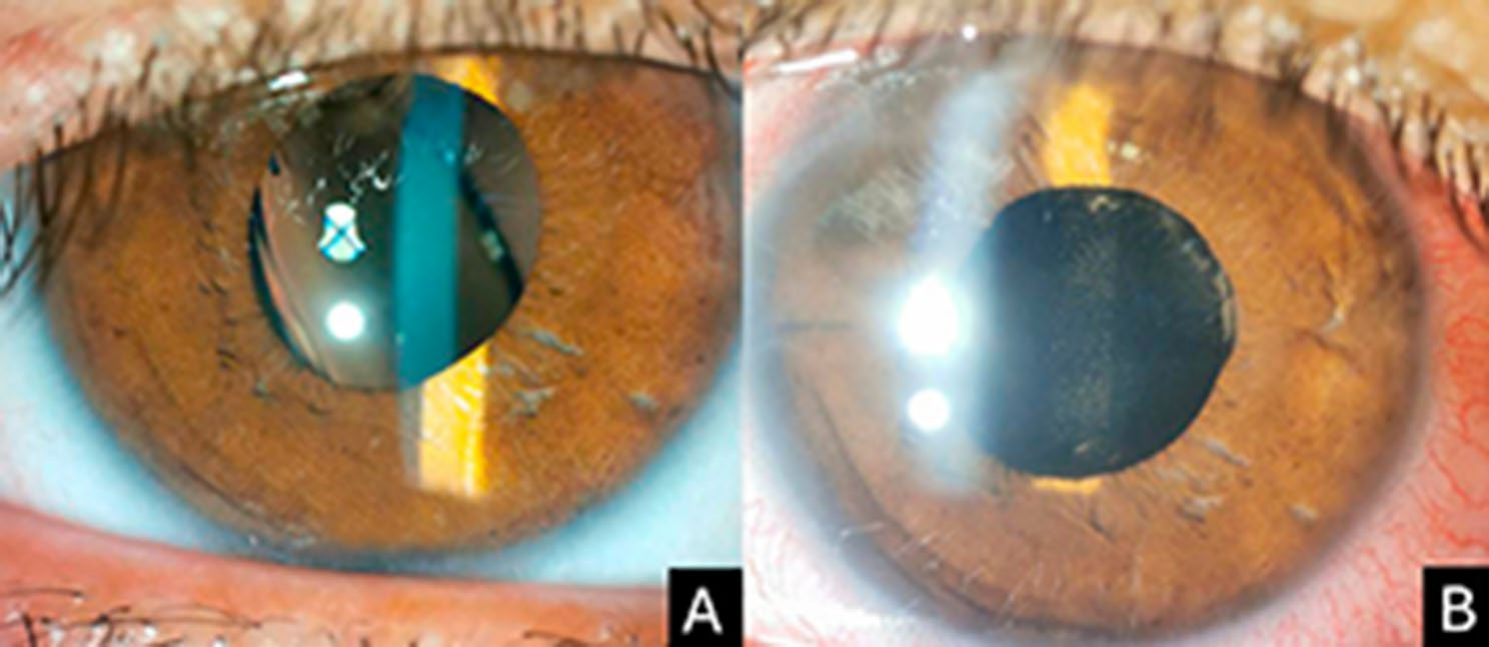
(**A**) A slit-lamp photograph shows an eye with a dislocated intraocular lens (IOL). (**B**) A slit-lamp photograph of the same eye obtained on the first postoperative day after retropupillary fixation of an iris-claw IOL

A total of 33 eyes of 32 patients was analyzed. The baseline characteristics are summarized in Table 1. Three eyes had a history of macula-on rhegmatogenous retinal detachment previously treated with pars plana vitrectomy. In all cases, retinal breaks were in the superior peripheral retina and gas tamponade was used. All eyes had a stable and attached retina at the time of IOL exchange surgery. The mean follow-up time was 10.18 ± 5.96 months (range, 6–25 months). The indications for IOL exchange included IOL dislocation into the posterior vitreous in nine eyes (27.3%), IOL opacification in four eyes (12.1%), and IOL subluxation into the anterior vitreous or posterior chamber in 20 eyes (60.6%) (Fig. 1). The main causes of posterior chamber IOL subluxation were a history of complicated cataract surgery with late in-the-bag IOL instability, and zonular weakness. All procedures included combined PPV, IOL removal, and retropupillary ICIOL fixation. The mean surgical time was 25.3 ± 4.5 min. Two patients (6.1%) developed intraoperative hyphema.

**Table 1:** Baseline characteristics before Iris claw intraocular lens fixation

Parameter	*N* = 33 patients
Gender - male, n (%)	24 (72.7%)
Age (years)	
Mean ± SD	68.3 ± 14.3
Ocular history,^*^ n (%)	
No previous ocular pathology	23 (69.6%)
Glaucoma	5 (15.2%)
Macula-on retinal detachment	3 (9.1%)
Non-proliferative diabetic retinopathy	2 (6.1%)
Ocular toxoplasmosis	1 (3.0%)
Previous surgeries	
Phacoemulsification	33 (100%)
Vitrectomy	3 (9.1%)

^*^Multiple answer - the sum of the percentages does not total 100.0%

SD, standard deviation; n, number

The VA, refraction, and corneal analysis are shown in Table 2. At 6 months postoperatively, the BCVA improved significantly from 0.73 ± 0.55 logMAR to 0.18 ± 0.24 logMAR (*p* < 0.001). Twenty-seven patients (81.8%) achieved a final BCVA between 20/20 and 20/40. The VA improved in 31 patients (93.9%), remained unchanged in two (6.1%), and deteriorated in none.

The final mean SE was − 0.40 ± 0.97 D compared to the preoperative 3.33 ± 6.29 D (*p* = 0.001). There was a significant increase in the proportion of eyes with a postoperative SE within ± 0.50 D of target (from 24.2% preoperatively to 66.7% postoperatively) and a reduction in eyes outside ± 2.0 (from 66.7% to 21.2%, *p* = 0.002) postoperatively (Table 2).

**Table 2:** Visual acuity, refraction and corneal analysis

Parameter	Preoperative value	Postoperative value	*p* value
BCVA (logMAR)			
Mean ± SD	0.73 ± 0.55	0.18 ± 0.24	< 0.001^a^
Median (IQR)	0.60 (0.35 to 0.95)	0.05 (0.00 to 0.30)	
Spherical equivalent			
Mean ± SD	3.33 ± 6.29	-0.40 ± 0.97	0.001^a^
Range (min to max)	-5.00 to 13.50	-3.00 to 1.50	
Spherical equivalent			
± 0.5 D	8 (24.2%)	22 (66.7)	0.002^b^
± 1.0 D	3 (9.1%)	4 (12.1)	
± 2.0 D	22 (66.7%)	7 (21.2)	
Astigmatism			
Mean ± SD	1.34 ± 1.85	1.36 ± 1.30	0.955^a^
Range (min to max)	6.00 to 3.00	5.00 to 0.00	
Axis			
With-the-rule	19 (57.6%)	18 (54.5)	0.362^b^
Against-the-rule	7 (21.2%)	11 (33.3)	
Oblique	7 (21.2%)	4 (12.1)	
Topography			
Flat K	42.07 ± 1.98	42.48 ± 2.66	0.449 ^a^
Steep K	43.91 ± 2.01	44.79 ± 1.34	0.033 ^a^
Astigmatism	1.83 ± 0.95	2.32 ± 1.47	0.259 ^a^

p – Mixed linear model (^a^) and McNemar test (^b^)

SD, standard deviation; BCVA, best-corrected visual acuity; logMAR, logarithm of the minimum angle of resolution; D, diopters; IQR, interquartile range

The mean final refractive astigmatism was 1.36 ± 1.30 D, which did not differ significantly from the preoperative value of 1.34 ± 1.85 D (*p* = 0.955). No statistically significant changes were observed in corneal topography during the postoperative period (Table 2). Regarding biometric data, the mean axial length was 25.15 ± 1.90 mm, the mean implanted IOL power was 16.17 ± 4.62 D, and the mean target refraction was − 0.72 ± 0.45 D. The mean SIA was 1.39 ± 0.93 D, and the mean prediction error was 0.28 ± 0.99 D.

The mean central corneal thickness was 562 ± 35.09 μm preoperatively and 558 ± 42.15 μm postoperatively (*p* = 0.122). A transient increase in pachymetry (< 5%) was observed in about one-third of eyes, with no persistent edema. The mean IOP was 15.85 ± 4.89 mmHg preoperatively and 14.72 ± 4.10 mmHg postoperatively (*p* = 0.563), with no significant change observed.

The postoperative complications included transient hyphema in three eyes (9.1%), one case of hypotony (3.0%) with spontaneous resolution, two cases of CME (6.1%) successfully treated with topical corticosteroids and nonsteroidal anti-inflammatory drugs, and two cases of ocular hypertension (6.1%) controlled with topical hypotensive agents. Two patients (6.1%) developed postoperative IOL disenclavation: one after horseback riding and one following blunt trauma with a tennis ball. The first was managed with an IOL exchange for a scleral-fixated Akreos lens with Gore-Tex sutures and the second with surgical re-enclavation of the Artisan IOL. No vitreous-related complications, such as retinal tears, retinal detachments, or endophthalmitis, and no clinically significant pigment dispersion syndrome or pigmentary glaucoma were observed during the follow-up period.

## Discussion

This study demonstrated that IOL exchange combining PPV with retropupillary fixation of an ICIOL provides favorable visual and refractive outcomes with a low complication rate. These findings support the growing use of retropupillary iris-claw IOLs as an effective option for aphakic eyes without capsular support.

The visual outcomes in our series showed a mean final BCVA of 0.18 ± 0.24 logMAR, with 93.9% of eyes showing improvement. These results are comparable to or better than those reported in previous studies on retropupillary ICIOL fixation, in which the mean BCVA values ranged from 0.27 to 0.64 logMAR [5, 9, 10, 13, 14], and similar to outcomes achieved with other fixation techniques, such as four-point scleral fixation and Carlevale lenses that ranged from 0.40 to 0.66 logMAR [8, 15]. The consistency of these findings highlights the safety and reproducibility of the ICIOL approach when performed using a standardized surgical technique.

The refractive analysis further reinforces the value of this procedure. The mean SE in this cohort was − 0.40 ± 0.97 D, which was near emmetropia and comparable to previous reports using retropupillary iris-claw IOLs, which showed mean final SE values ranging from + 0.10 to -0.70 [10, 14, 16, 17]. Forlini and Bedi [13] reported a worse final SE of − 1.50 ± 1.15 D, likely influenced by the multiple procedures performed during the same surgical session, including complicated cataract extraction, PPV, and IOL fixation, which probably resulted in greater ocular manipulation. Outcomes with alternative fixation methods, including four-point scleral fixation, which demonstrated SEs ranging from − 0.57 D to − 1.29 D [8, 18], as well as Carlevale IOL implantation with SE values ranging from − 0.30 D to − 0.71 [7, 19], have shown similar or less favorable results. These findings indicate that the ICIOL approach provides favorable refractive results compared to other secondary IOL techniques.

The mean final postoperative refractive astigmatism in our series was 1.36 ± 1.30 D, which was comparable to those reported with the same technique, 1.85 ± 2.16 D [14] and 1.08 ± 0.43 D [20], and more favorable than 3.64 ± 3.34 D [9]. The poorer outcome in the last study was attributed to the use of corneoscleral incisions, which tend to induce greater refractive changes than a scleral tunnel 2 mm from the limbus.

In the current study, the mean SIA was 1.39 ± 0.93 D, similar to other series with retropupillary iris-claw IOLs that have reported mean SIAs of 1.72 ± 0.96 D [5] and 1.12 ± 0.85 D [21]. Methodologic differences must be considered: Seknazi et al. [5] did not specify the calculation method, whereas Kristianslund et al. [21] used vector analysis, in contrast to the Hill calculator used in the present series. Hernandez Martinez and Gonzalez [17] compared SIAs induced by corneal incisions (2.49 ± 1.36 D), which was significantly higher than our findings, with scleral tunnel incisions, which showed a lower mean SIA of 0.73 ± 0.62 D. Pardini et al. reported a mean SIA of 1.59 ± 0.41 D with four-point scleral fixation, similar to the current results [22]. More recently, another study compared the Carlevale IOL with retropupillary iris-claw implantation and reported a significantly lower mean SIA in the Carlevale group (0.3 ± 1.8 D) compared to the iris-claw group (0.8 ± 2.1 D) (*p* = 0.01) [15]. These results reinforced the influence of the incision type, fixation site, and IOL design on postoperative astigmatism and supported the role of retropupillary iris-claw fixation with scleral tunnel incisions as an effective approach compared with other fixation techniques.

The prediction error was − 0.28 ± 0.99 D, which agreed with other series using retropupillary iris-claw fixation ranging from 0.26 to 0.99 D [5, 16, 21, 23]. Comparable results also have been reported with alternative approaches, such as four-point scleral fixation with Gore-Tex sutures (− 0.19 ± 0.72 D) [8] and the Carlevale IOL (− 0.24 ± 0.81 [19] and − 0.27 ± 0.78) [23]. This reproducibility suggested that surgeons have learned to adjust IOL power relative to the fixation site, minimizing refractive surprises. Nevertheless, a direct comparison between the Carlevale and Artisan IOLs revealed a significant difference in the prediction error, with a mean of -0.46 D for the Carlevale IOL and 0.08 D for the Artisan IOL (*p* = 0.019) [24]. This finding is relevant because with retropupillary iris-claw implantation, the lens is consistently positioned in the iris plane, reducing variability in the effective lens position and, consequently, minimizing biometric calculation errors. This contrasts with scleral fixation techniques, in which IOL positioning can vary considerably and greater uncertainty can be introduced in the power calculation. In our series, the use of an A-constant of 116.9 resulted in accurate outcomes, consistent with previous recommendations [9, 11].

The SRK/T formula, developed by Sanders, Retzlaff, and Kraff [25], was used for IOL power calculation, in accordance with previous publications on retropupillary iris-claw IOL fixation, based on institutional preference and its well-established performance in myopic eyes. Several studies evaluating retropupillary ICIOL implantation have adopted the SRK/T formula and reported reliable refractive outcomes [9, 11, 13–16, 20, 21, 26]. Although widely validated, SRK/T has known limitations related to the prediction of effective lens position, a key source of postoperative refractive error, particularly in eyes with unusual axial lengths or less predictable postoperative IOL position [27]. Alternative formulas such as Haigis or Barrett Universal II could also be considered, particularly in eyes with longer axial length, such as in our cohort (mean axial length, 25.15 mm). Nevertheless, despite these theoretical limitations, the refractive outcomes achieved in our series were favorable and comparable to those reported in the literature, supporting the reliability of the selected formula in this clinical setting.

In our study, the mean duration of retropupillary iris-claw IOL implantation combined with PPV was 25.3 min, which agreed with previous reports describing retropupillary iris implantation as a quick and efficient procedure [4]. Czajka et al. and Bontemps et al. reported a mean surgical time of 40 min in a multicenter study [4, 15], which likely reflects variability across participating centers. Nevertheless, this surgical time compares favorably with other secondary IOL implantation techniques, such as the Carlevale FIL-SSF scleral-fixated IOL, which averaged between 53.4 and 72 min [6, 7] and the Akreos AO60 IOL fixated with Gore-Tex sutures, which required approximately 72 min in the series by Pardini et al. [8]. These comparisons were made with techniques involving similar procedural complexity, including IOL exchange combined with pars plana vitrectomy and secondary IOL fixation. Furthermore, a meta-analysis comparing the Yamane technique and scleral-sutured fixation reported mean durations ranging from 12.3 to 77.3 min for the Yamane approach and 21.8 to 107.4 min for scleral-sutured fixation [28].

Our complication rates were consistent with those in published series [8, 10, 29]. Importantly, no vitreous-related complications such as retinal detachments, retinal tears, or endophthalmitis occurred, likely reflecting the routine use of PPV in all cases, which reduces vitreous traction and associated risks [30]. Studies in which posterior vitrectomy was performed in all cases reported no vitreous-related complications [26, 31]. In contrast, studies that performed only anterior vitrectomy and/or posterior vitrectomy in selected cases reported retinal detachment as a postoperative complication [9, 10, 32]. Although anterior vitrectomy may be sufficient in selected cases, a complete PPV was routinely performed in our series to facilitate safe IOL explantation and fixation and to minimize vitreoretinal complications. Despite this approach, postoperative inflammation was mild and transient, with no cases of persistent or severe inflammatory reaction.

Although some authors consider the risk of iris-claw intraocular lens dislocation after retropupillary implantation to be low [14, 16], previous studies have reported disenclavation or dislocation rates of up to 9.09% [5, 11, 13]. In our series, we observed two cases of enclavation failure, both related to external trauma rather than insufficient tissue grasping. The first occurred when a patient was horseback riding and the second after blunt ocular trauma with a tennis ball. Both cases required reoperation, one managed with scleral fixation of an Akreos IOL using Gore-Tex sutures, and the other with re-enclavation of the Artisan IOL. These findings highlight the importance of addressing patient’s lifestyle and hobbies during preoperative assessment. For individuals involved in impact sports, surgeons should carefully weigh the risks and benefits of retropupillary iris-claw implantation, as disenclavation or dislocation, though uncommon, can still occur.

No cases of reverse pupillary block developed in this series, and a peripheral iridectomy was not routinely performed using the vitrector probe during surgery. When the ICIOL is fixated on the anterior surface of the iris, the risk of pupillary block is higher, and performing a peripheral iridectomy is generally recommended [10, 31–33]. Although some studies have described performing iridectomy even with retropupillary fixation, most authors have reported satisfactory outcomes without this additional step [5, 11, 13, 16]. Nonetheless, long-term postoperative follow-up remains important, as pupillary block may develop months or even years after surgery.

Another concern with ICIOL implantation is endothelial safety. Although endothelial cell loss ranging from 5% to 12% has been reported after both anterior and retropupillary Artisan fixation [14, 26, 29, 33], no significant difference was found between the two positions. In our series, no clinical signs of corneal decompensation were observed, and pachymetry remained stable throughout follow-up period. Endothelial cell count data were not consistently available due to the retrospective design of the study; therefore, corneal pachymetry and clinical signs of corneal decompensation were used as surrogate markers of endothelial safety and remained stable. Future prospective studies should include endothelial cell density analysis.

The strengths of this study were the analysis of IOL exchange with retropupillary iris-claw fixation through a comprehensive refractive and anatomic evaluation. Beyond the VA, we assessed the SE, final refractive astigmatism, SIA, and prediction error in a homogeneous cohort, together with corneal pachymetry, tomography, and biometry. This level of detail in refractive analysis has not been described previously, and it underscores the reliability and reproducibility of this approach. Additionally, the standardized single-surgeon technique reduces variability, further reinforcing the robustness of our results.

The limitations of this study include its retrospective design, relatively modest sample size, and short follow-up period, which might limit the generalizability of the findings and the ability to detect rare complications and may introduce survival bias. In addition, preoperative refractive measurements may have been influenced by the position of the dislocated IOL. Although subgroup analysis according to IOL position (posterior vitreous cavity, anterior vitreous, or opacified IOL) could provide additional insight, the limited number of eyes in each subgroup precluded a robust statistical comparison. These issues should be addressed in future prospective studies with larger and more homogeneous cohorts.

In conclusion, IOL exchange with combined PPV and retropupillary iris-claw fixation is an efficient and reproducible technique that provides good visual rehabilitation, reliable refractive predictability, and an acceptable safety profile. By demonstrating consistent outcomes across multiple refractive parameters, this study highlights the clinical value of this approach and supports its role as a valuable option in the management of aphakia without capsular support.

## Supplementary Information

Below is the link to the electronic supplementary material.

Supplementary Material 1

## Data Availability

The datasets used and/or analyzed during the current study are available from the corresponding author on reasonable request.

## Abbreviations

ACIOL: Anterior-chamber IOL
CME: Cystoid macular edema
D: Diopter
ICIOL: Iris claw intraocular lens
IOL: Intraocular lens
IOP: Intraocular pressure
logMAR BCVA: Logarithm of the minimum angle of resolution best-corrected visual acuity
PCIOL: Posterior-chamber IOL
PPV: Pars plana vitrectomy
SE: Spherical equivalent
SIA: Surgically induced astigmatism
